# A three-lncRNA signature predicts overall survival and disease-free survival in patients with esophageal squamous cell carcinoma

**DOI:** 10.1186/s12885-018-4058-6

**Published:** 2018-02-06

**Authors:** Guo-Wei Huang, Yu-Jie Xue, Zhi-Yong Wu, Xiu-E Xu, Jian-Yi Wu, Hui-Hui Cao, Ying Zhu, Jian-Zhong He, Chun-Quan Li, En-Min Li, Li-Yan Xu

**Affiliations:** 1 Institute of Oncologic Pathology, Shantou University Medical College, No. 22, Xinling Road, Shantou, Guangdong 515041 China; 2 The Key Laboratory of Molecular Biology for High Cancer Incidence Coastal Chaoshan Area, Shantou University Medical College, Shantou, 515041 Guangdong People’s Republic of China; 3 Department of Biochemistry and Molecular Biology, Shantou University Medical College, Shantou, 515041 Guangdong People’s Republic of China; 4 Departments of Oncology Surgery, Shantou Central Hospital, Affiliated Shantou Hospital of Sun Yat-sen University, Shantou, 515041 Guangdong People’s Republic of China; 5 School of Medical Informatics, Daqing Campus, Harbin Medical University, Daqing, 163319 Heilongjiang People’s Republic of China

**Keywords:** LncRNAs, *RP11-366H4.1.1*, *LINC00460*, *AC093850.2*, Signature, Esophageal squamous cell carcinoma, Overall survival, Disease-free survival

## Abstract

**Background:**

Increasing evidence shows that dysregulated long non-coding RNAs (lncRNAs) can serve as potential biomarkers for cancer prognosis. However, lncRNA signatures, as potential prognostic biomarkers for esophageal squamous cell carcinoma (ESCC), have been seldom reported.

**Methods:**

Based on our previous transcriptome RNA sequencing analysis from 15 paired ESCC tissues and adjacent normal tissues, we selected 10 lncRNAs with high score rank and characterized the expression of those lncRNAs, by qRT-PCR, in 138 ESCC and paired adjacent normal samples. These 138 patients were divided randomly into training (*n* = 77) and test (*n* = 59) groups. A prognostic signature of lncRNAs was identified in the training group and validated in the test group and in an independent cohort (*n* = 119). Multivariable Cox regression analysis evaluated the independence of the signature in overall survival (OS) and disease-free survival (DFS) prediction. GO and KEGG pathway analysis, combined with cell transwell and proliferation assays, are applied to explore the function of the three lncRNAs.

**Results:**

A novel three-lncRNA signature, comprised of *RP11-366H4.1.1* (ENSG00000248370), *LINC00460* (ENSG00000233532) and *AC093850.2* (ENSG00000230838), was identified. The signature classified patients into high-risk and low-risk groups with different overall survival (OS) and disease-free survival (DFS). For the training group, median OS: 23.1 months vs. 39.1 months, *P* < 0.001; median DFS: 15.2 months vs. 33.3 months, *P* < 0.001. For the test group, median OS: 23 months vs. 59 months, *P* < 0.001; median DFS: 16.4 months vs. 50.8 months, *P* < 0.001. For the independent cohort, median OS: 22.4 months vs. 60.4 months, *P* < 0.001). The signature indicates that patients in the high-risk group show poor OS and DFS, whereas patients with a low-risk group show significantly better outcome. The independence of the signature was validated by multivariable Cox regression analysis. GO and KEGG pathway analysis for 588 protein-coding genes-associated with the three lncRNAs indicated that the three lncRNAs were involved in tumorigenesis. In vitro assays further demonstrated that the three lncRNAs promoted the migration and proliferation of ESCC cells.

**Conclusions:**

The three-lncRNA signature is a novel and potential predictor of OS and DFS for patients with ESCC.

**Electronic supplementary material:**

The online version of this article (10.1186/s12885-018-4058-6) contains supplementary material, which is available to authorized users.

## Background

In recent years, advances in the depth and quality of transcriptome sequencing have allowed for the rapid discovery of lncRNAs, and accumulated evidence shows that lncRNAs are functional transcripts rather than biological noise. LncRNAs regulate diverse cellular processes, including chromatin modification, transcription initiation, and co- and post-transcriptional regulation [1]. A large number of studies has begun to expound the roles of lncRNAs in different biological systems, such as the reproductive, metabolic, and immune systems [2]. The prognostic power of lncRNA signatures has been investigated in various cancers, including glioblastoma multiform [3], colorectal cancer [4], non-small cell lung cancer [5] and esophageal cancer [6].

Esophageal cancer is one of the most common malignant tumors worldwide [7], nearly 450,000 new cases are diagnosed annually, and around 70% of the cases occur in China. Among the various histological subtypes of esophageal cancer, esophageal squamous cell carcinoma (ESCC) is the principal form in the vast majority of cases [8]. Its overall 5-year survival is less than 20%,due to the difficulty of early detection and frequent metastatic recurrence [9, 10]. For ESCC, the clinical staging system (pTNM) is the main prognostic indicator, but it has limited capacity in clinical practice. Increasing evidence has shown that mRNA or miRNA signatures are strong predictors of survival in patients with ESCC [11–13], and whether lncRNA signatures have similar prognostic power in esophageal cancer has drawn recent interest. Tong et al. selected ten lncRNAs based on a previous study and identified lncRNA *POU3F3* in plasma as a novel biomarker for diagnosis of ESCC [14]. Li et al., using a lncRNA expression profile microarray, revealed a three-lncRNA signature associated with the survival of ESCC patients [6], and our research group combined protein-coding genes with long non-coding RNA to predict prognosis for patients with ESCC as a novel clinical multi-dimensional signature [15], suggesting that lncRNAs can be promising prognostic biomarkers for ESCC for use in the clinic.

In a previous study, we reported lncRNA expression profiles in 15 paired ESCC tissues and adjacent non-tumor tissues via transcriptome RNA sequencing, and further developed a method, denoted URW-LPE, for Unsupervised Random Walk with each dysregulated LncRNA/PCG, to identify novel potential functional lncRNAs. A seed composed of each dysregulated lncRNA/PCG (protein coding gene), combined with an edge composed of an extended co-expression relation, was used as a random walk. Differentially-expressed lncRNAs and PCGs in ESCC were used to construct an extended lncRNA-PCG co-expression network and the random walk was run for the network and the fold change (FC) values of each node on the network was considered as the initial probability vector. Thus, each lncRNA in the network would be given an URWScore value and lncRNAs with the higher URWScore value would be expected to possess more important biological functions in ESCC [16]. However, whether the potential lncRNAs could be used as prognostic biomarkers for ESCC needs to be further determined. In the present study, from the previously identified lncRNA biomarkers, we selected 10 higher-ranking lncRNAs, and then detected their expression using quantitative RT-PCR (qRT-PCR) in 138 patients with ESCC. Finally, we built another three-lncRNA signature (*RP11-366H4.1.1*, *LINC00460* and *AC093850.2*) that was highly associated with the overall survival and disease-free survival of patients with ESCC, and further validated its prognostic value in an independent cohort of 119 patients (GSE53624) from the Gene Expression Omnibus (GEO) database [6].

## Methods

### Patients and tissue specimens

We collected paired tumor and adjacent non-tumor tissues from 138 patients with ESCC (between 2007 and 2009), from the Department of Oncological Surgery of the Central Hospital of Shantou City, P.R. China. Cases were selected in this study only if a follow-up was obtained and clinical data were available. The follow-up for patients after esophageal resection was continued until their deaths and only patients that died from ESCC were included in the tumor-related deaths. Patients, suffering from severe post-operative complications, other tumors or died of other causes were excluded. This study was approved by the Ethical Committee of the Central Hospital of Shantou City and the Medical College of Shantou University, and written informed consent was obtained from all surgical patients to use resected samples and clinical data for research. The tissue specimens were snap frozen in liquid nitrogen shortly after resection and stored at − 80 °C until RNA extraction.

### Cell lines and culture conditions

Human esophageal cancer cell lines KYSE150, KYSE180, KYSE450, KYSE70, KYSE140 and TE3 were kindly provided by Dr. Ming-Zhou Guo (Chinese PLA General Hospital, Beijing, China) and grown in RPMI 1640 medium (Invitrogen, California, USA), with both media supplemented with 10% FBS (Invitrogen, California, USA). The human immortalized esophageal cell line NE2 was kindly provided by Professor Sai-Wah Tsao (University of Hong Kong, China) and grown in defined keratinocyte serum-free medium (Gibco, Grand Island, NY, USA) and Cascade Biologics® EpiLife® (Life Technologies, Grand Island, NY, USA) in a 1:1 mixture. All cell lines were cultured at 37 °C in 5% CO2 and 95% air.

### RNA extraction

Human samples or cell lines were lysed using TRIzol® (15596-018, Life Technologies, Carlsbad, CA, USA) and total RNA was released and further purified with a PureLinkTM RNA Mini Kit (12183018A, Life Technologies, Carlsbad, CA, USA) according to the manufacturer’s protocol. The purity and concentration of RNA were determined by OD260/280 using spectrophotometer (NanoDrop ND-2000).

### Quantitative RT-PCR (qRT-PCR)

For qRT-PCR, the reverse transcription (RT) reactions were carried out with a PrimeScriptTM RT reagent kit with gDNA Eraser (RR047A, TaKaRa, Dalian, China) according to the manufacture’s protocol. Reverse transcriptase reactions contained 1 μg total RNA. The 20 μl RT reaction mixture was incubated in a 2720 Thermal Cycler (Applied Biosystems). Quantitative PCR reactions were then performed on an ABI 7500 with SYBR® Premix Ex TaqTM (RR420A, TaKaRa, Dalian, China) in a 20 μl reaction volume, which also contained 2 μl cDNA and 0.8 μl PCR primer mix (forward and reverse primers at a final concentration of 0.2 μM each). The reactions were incubated at 95 °C for 30 s, followed by 40 cycles of 95 °C for 5 s, and 60 °C for 34 s. The Ct value of each candidate lncRNA was then normalized to the expression value of β-actin. Relative expression levels of the lncRNAs were calculated using the 2^-ΔCt^ method. Specimens that had no amplification within 40 cycles were deleted. Sequences of primers for qRT-PCR of the lncRNAs are listed in Additional file 1: Table S1.

### SiRNA transfection

Cells were transfected with siRNAs against RP11-366H4.1.1, LINC00460 and AC093850.2, with scrambled siRNA used as a negative control. The procedures for siRNA transfection were performed according to the X-tremeGENE siRNA transfection reagent instructions (Sigma-Aldrich, St. Louis, MO). The sequences for RP11-366H4.1.1 were sense: 5’-ACACACAUCCUAGUUCUUUdtdt-3′, and antisense: 5’-AAAGAAC UAGGAUGUGUGUdtdt-3′. The sequences for LINC00460 were sense: 5’-GUCACCCCGAUUUAUGUUAdtdt-3′, and antisense: 5’-UAACAUAAAUCGGGGUGACdtdt-3′. The sequences for AC093850.2 were 5’-GGACAAUGAAGACUGAACUdtdt-3′, and antisense: 5’-AGUUCAGUCUUCAUUGUCCdtdt-3′. The negative control siRNA was sense: 5’-UUCUCCGAACGUGUCACGdtdt-3′, and antisense: 5’-CGUGACACG UUCGGAGAAdtdt-3′.

### Cell migration and colony formation assays

After *RP11-366H4.1.1*, *LINC460* or *AC093850.2* was subjected to individual knockdown, KYSE150 or KYSE70 cell migration and colony formation assays were performed as previously described [16]. Briefly, at 24 h post transfection, cells were starved for 12 h with serum-free medium (Invitrogen, California, USA) and then 5 × 10^4^ cells were plated in serum-free medium in the upper well of a transwell chamber (24-well insert; pore size, 8 μm; BD Biosciences, Franklin Lakes, NJ, USA), and the lower chamber containing medium with 10% FBS. After 48 h, cells in the top chamber were removed with a cotton swab and only cells that migrated through the pores were fixed and stained in haematoxylin solution (Sigma-Aldrich, St. Louis, MO, USA) and counted. For colony formation, 500 cells per well in 24-well plate were incubated in medium supplemented with 10% FBS for ten days, and then colonies were stained with haematoxylin solution and observed.

### URWScore and bioinformatics analysis

The URW-LPE method has been previously described in detail [16]. Briefly, a seed composed of each dysregulated lncRNA/PCG (protein coding gene), combined with an edge composed of an extended co-expression relation, was used as a random walk. Differentially-expressed lncRNAs and PCGs in ESCC were used to construct an extended lncRNA-PCG co-expression network and the random walk was run for the network and the fold change (FC) values of each node on the network was regarded as the initial probability vector. The random walk was represented according to the formula: p^t + 1^ = (1-r) Wp^t^ + rp^0^. W is represented by the adjacency matrix in the lncRNA-PCG co-expression network, p^t^ is a vector representing the probability of the corresponding lncRNA /PCG nodes at step t and p^0^ is used as the initial probability vector. Thus, each lncRNA in the network would be given an URWScore value and lncRNAs with a higher URWScore value may possess more important biological functions in ESCC. In the lncRNA-PCG co-expression network, protein-coding genes highly associated with the higher URWScoring lncRNAs (Pearson correlation coefficient > 0.40, *P* < 0.05) were selected. The association of the lncRNAs with potential protein-coding genes was visualized by Cytoscape_v2.8.3 software [17]. GO (Gene Ontology) and KEGG (Kyoto Encyclopedia of Genes and Genomes) pathway function enrichment analyses for the co-expressed protein-coding genes were performed according to the DAVID database on line (https://david.ncifcrf.gov/) [18].

### Statistical analysis

The 138 specimens were randomly separated into a training set (*n* = 77) and test set (*n* = 61). A multivariable Cox regression model in the training set, including age, gender, histologic grade, invasive depth, lymph node metastasis and therapies, was constructed [19]. Comparisons between the two sets for clinicopathological characteristics was performed using the *t*-test, Fisher’s exact test and chi-squared test. Comparisons of the relative expression between tumor and paired adjacent normal tissues were performed using paired a *t*-test. Overall survival (OS) was measured from the date of surgery to death or the latest follow-up. Disease-free survival (DFS) was measured from the date of surgery to the first occurrence of any of the following events, including recurrence, distant metastasis or death from any cause without documentation of a cancer-related event [20]. The optimal cut-off point of lncRNA expression (2^-ΔΔCt^, ΔΔCt = ΔCt tumor – ΔCt normal, ΔCt = Ct (selected lncRNA) – Ct (β-actin)) and risk score were assessed by the X-tile program [21]. According to the cutoff value, the relative levels of lncRNAs from 138 paired ESCC samples and adjacent normal tissues was divided into high or low expression groups using X-tile and then probabilities of OS and DFS patients with ESCC were calculated by Kaplan-Meier analysis and compared using the log-rank test with SPSS19.0 (IBM, Armonk, New York, USA). A two-tailed *P*-value less than 0.05 was considered to have statistical significance. All analyses were performed using SPSS 19.0 (IBM, Armonk, New York, USA) for Windows.

## Results

### The selection of lncRNAs with higher URWScores

In a previous study, we identified 275 statistically significant potential lncRNAs through URW-LPE [16]. The higher URWScore lncRNAs may possess more important biological functions in ESCC. Based on the URWScore, 10 lncRNAs with higher URWScore value were selected (Table 1) to identify a lncRNA signature associated with overall survival and disease-free survival of patients with esophageal cancer.

**Table 1:** Basic information about the 10 lncRNAs

Rank	Ensemble ID	Gene symbol	LogFC	Random walk score
3	ENSG00000259756	RP11-625H11.2.1	11.92339	0.000597015
10	ENSG00000248370	RP11-366H4.1.1	11.92339	0.000562804
43	ENSG00000233532	LINC00460	6.30885	0.000503077
59	ENSG00000230838	AC093850.2.1	6.987091	0.000485158
101	ENSG00000242147	RP13-463 N16.6.1	6.562914	0.000454164
162	ENSG00000235237	RP1-151B14.6.1	2.846653	0.000415793
184	ENSG00000236427	RP11-107 M16.2.1	−4.56484	0.000399876
196	ENSG00000258689	LINC01269	−4.35277	0.000395749
222	ENSG00000223485	RP11-417E7.1.1	5.63628	0.000382129
229	ENSG00000232335	RP11-435D7.3.1	2.896612	0.000379418

### Validation of lncRNA expression levels by qRT-PCR

**Fig. 1:**
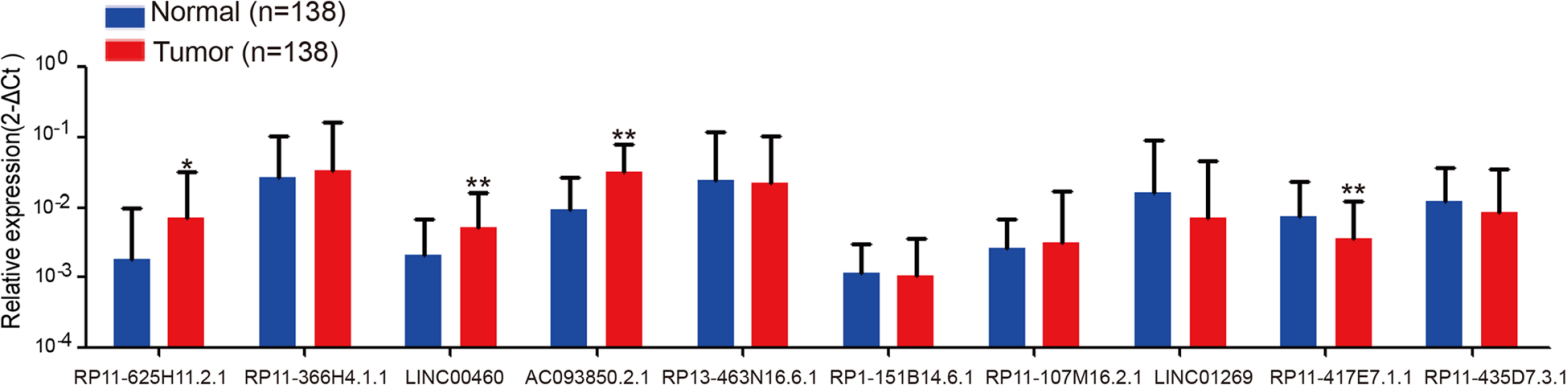
LncRNAs were measured by qRT-PCR. RNA was extracted from 138 paired ESCC tissues and adjacent normal tissues and ten selected lncRNAs were quantified by qRT-PCR according to the 2^-∆Ct^ method. β-Actin was selected as an internal control. Triplicate samples of each were prepared. The data are represented by the mean ± standard error (SE). *P < 0.05, **P < 0.01

To further characterize the lncRNAs, we selected 10 lncRNAs with higher URW Score, 8 of them were up-regulated in tumor tissues, the other 2 were down-regulated (Table 1). Then we performed qRT-PCR to investigate lncRNA expression level in the 138 paired ESCC tissues. Results of the paired *t*-test showed that *RP11-625H11.2.1*, *RP11-417E7.1.1*, *LINC00460* and *AC093850.2* expressions were increased in ESCC samples, with only *RP11-417E7.1.1* expression opposing the results of RNA transcriptome sequencing (Fig. 1).

### Identification of three lncRNAs associated with OS and DFS from the training set

**Fig. 2:**
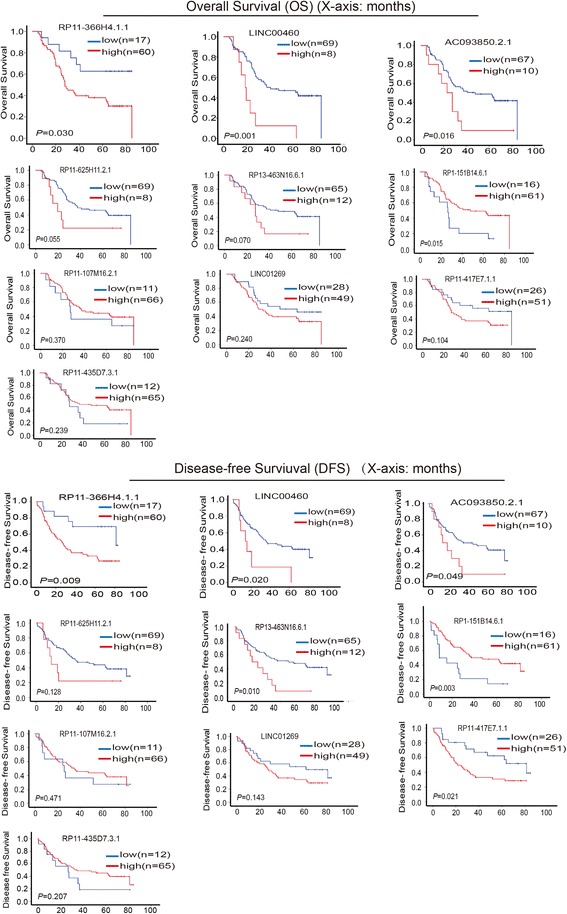
Kaplan-Meier analysis for the levels of 10 selected lncRNAs with the association with overall survival and disease-free survival, of patients with ESCC, in the training set (*n* = 77). *P*-values were calculated by the log-rank test. OS, overall survival; DFS, disease-free survival. Y-axis: OS or DFS; X-axis: Time (months)

For prognostic signature analysis, the 138 specimens were randomly separated into a training set (*n* = 77) and test set (*n* = 61). There were no significant differences in clinicopathologic characteristics between the two sets (Additional file 1: Table S2). In the training set, the relative levels of each of 10 selected lncRNAs were individually measured by real-time RT-PCR and then divided into a high or low expression group for each of lncRNAs according to the X-tile program [21] and Kaplan-Meier analysis was performed for overall survival (OS) time or disease-free survival (DFS) time for patients with ESCC. The results showed that *RP11-366H4.1.1*, *LINC00460* and *AC093850.2* were closely associated both with OS and DFS among ESCC patients (Fig. 2), with patients expressing higher levels of *AC093850.2*, *LINC00460* and *RP11-366H4.1.1* tending to have shorter survival time and earlier recurrence. However, patients expressing higher levels of *RP1-151B14.6.1* tended to have longer survival time and later recurrence. The other lncRNAs had no statistical significance between expression and OS or DFS in the training set (Fig. 2).

### Establishment and validation of a three-lncRNA signature for OS

**Fig. 3:**
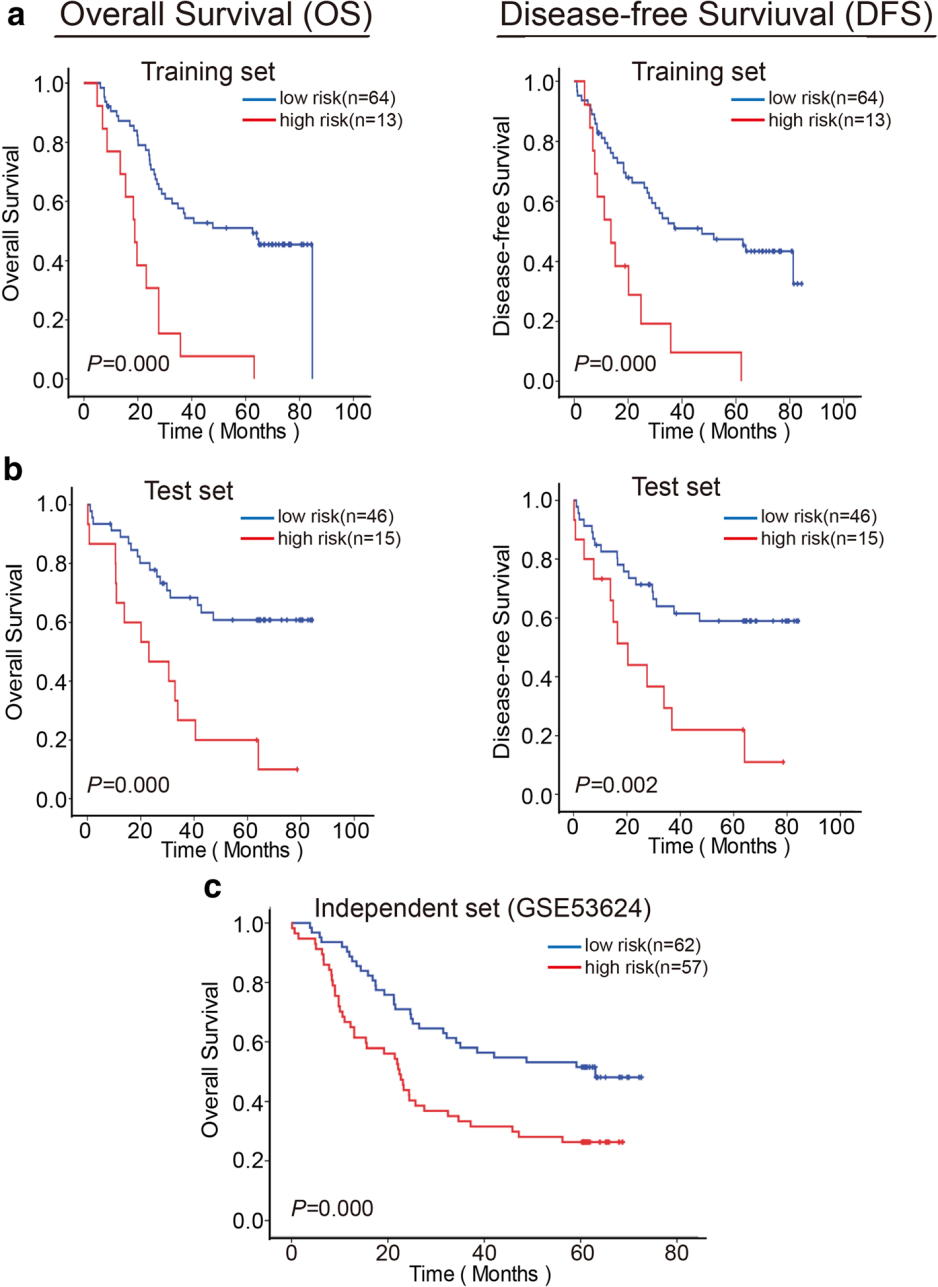
The three-lncRNA signature predicts overall survival and disease-free survival of patients with ESCC. Kaplan–Meier survival curves of patients are classified into high- and low-risk groups using the three-lncRNA signature in the (**a**) training set (*n* = 77), (**b**) test set (*n* = 61) and (**c**) independent set (*n* = 119). *P*-values were calculated by the log-rank test. OS, overall survival; DFS, disease-free survival

We selected *AC093850.2*, *LINC00460* and *RP11-366H4.1.1* to build a multivariable Cox regression model in the training set. Risk score of the predictive model for OS was obtained as follows: (0.882 × *AC093850.2*) + (1.219 × *LINC00460*) + (0.921 × *RP11-366H4.1.1*). The cut-off point was 48.48, as assessed by the X-tile program [21], and then the training set was divided into a high-risk group (*n* = 13) and a low-risk group (*n* = 64). Median OS in the high-risk group was significantly lower than that in low-risk group (23.1 months vs. 39.1 months, *P* < 0.001) (Fig. 3a, *left*). To test the value of the three-lncRNA signature, we used the same predictive model and threshold to divide the test set into high-risk groups (*n* = 15) and low-risk groups (*n* = 46). Median OS in the high-risk group was also lower than that in low-risk group (23 months vs. 59 months, *P* < 0.001) (Fig. 3b, *left*). To test the power of the prognosis signature further, we used an independent ESCC dataset (GSE53624) and acquired similar results (median OS: 22.4 months in the high-risk group vs. 60.4 months in the low-risk group, *P* < 0.001) (Fig. 3c).

### Confirmation of predictive value of the three-lncRNA signature for DFS

To confirm whether the three-lncRNA signature for OS can be used for predicting DFS, the same analysis was performed in the training set and test set. The three-lncRNA signature showed similar results in both sets (training set median DFS: 15.2 months in the high-risk group vs. 33.3 months in the low-risk group, *P* < 0.001; test set median DFS: 16.4 months in the high-risk group vs. 50.8 months in the low-risk group, *P* < 0.001) (Fig. 3a and b, *right*), demonstrating its prognostic capability to predict recurrence risk.

### The three-lncRNA signature is independent of clinical characteristics for survival prediction and recurrence prediction

Multivariable Cox regression analysis from the training set and test set (for OS: high-risk group vs. low-risk group, HR = 2.204, 95% CI 1.102–4.409, *P* = 0.025 in the training set; HR = 4.045, 95% CI 1.719–9.520, *P* = 0.001 in the test set; for DFS: high-risk group vs. low-risk group, HR = 1.959, 95% CI 1.058–3.628, *P* = 0.032 in the training set; HR = 3.269, 95% CI 1.501–7.118, *P* = 0.003 in the test set) (Table 2) showed that the prognostic power of the three-lncRNA signature risk score for prediction of survival was indeed independent of these clinical characteristics for prediction of survival and recurrence. Results showed lymph node metastasis (N stage) is an independent prognostic factor, too.

**Table 2:** The clinicopathological characteristics of patients with ESCC in the two datasets

Clinical and pathological indexes	Training set					Test set				
	Case No.	5-year OS (%)	P*	5-year DFS(%)	P*	Case No.	5-year OS (%)	P*	5-year DFS(%)	P*
Specimens	77	43.4		41.2		61	50.2		41.2	
Mean age	58					58				
Age (year)			0.959		0.964			0.017		0.014
< 58	45	41.9		41		35	63.2		62.9	
≥ 58	32	41.9		38.6		26	34.6		34.6	
Gender			0.978		0.464			0.995		0.995
Male	58	40.3		43.2		48	51.6		46.2	
Female	19	52.1		35.5		13	46.2		46.2	
Tumor location										
upper	7	57.1	0.354	71.4	0.247	5	20.0	0.317	20.0	0.125
middle	26	34.6		32.8		22	49.2		44.3	
lower	44	46.9		41.4		34	5		58.7	
Histologic grade			0.093		0.121			0.298		0.222
G1	17	47.5		47.5		8	72.9		56.3	
G2	55	44.5		39.5		46	49.4		48.8	
G3	5	20.0		20.0		7	28.6		28.6	
Invasive depth			0.052		0.183			0.427		0.407
T1	5			60.0		3	66.7		50.0	
T2	9	22.2		22.2		7	28.6		28.6	
T3	62	42.6		41.9		51	52.4		50.2	
T4	1	0.0				0	–		–	
Lymph node metastasis			0.002		0.007			0.115		0.120
N-neg	42	58.5		56.2		29	61.9		57.9	
N-pos	35	24.5		20.8		32	38.6		37.3	
pTNM-stage			0.002		0.077			0.262		0.371
I	11	71.6		61.4		5	75.0		75.0	
II	36	52.8		49.8		27	55.6		47.5	
III	30	25.4		20.8		29	39.0		42.0	
Therapies			0.354		0.522			0.560		0.815
Only Surgery	56	36.1		34.4		38	52.5		49.3	
Postoperative Chemotherapy	6	50.0		50.0		19	66.7		55.6	
Postoperative Radiotherapy	9	66.7		66.7		12	33.3		33.0	
Others^a^	6	66.7		50.0		2	50.0		50.0	

All patients underwent surgical treatment. OS: overall survival; DFS: disease free survival

Test set: Preoperative Chemotherapy and Radiochemotherapy (1 cases), Preoperative and Postoperative Chemotherapy (1 cases)

*Log-rank test of Kaplan Meier method; *P* < 0.05 was considered significant

^a^Training set: Postoperative Chemotherapy and Radiochemotherapy (5 cases), Preoperative and Postoperative Chemotherapy (1 cases)

### Function of the three-lncRNA signature

**Fig. 4:**
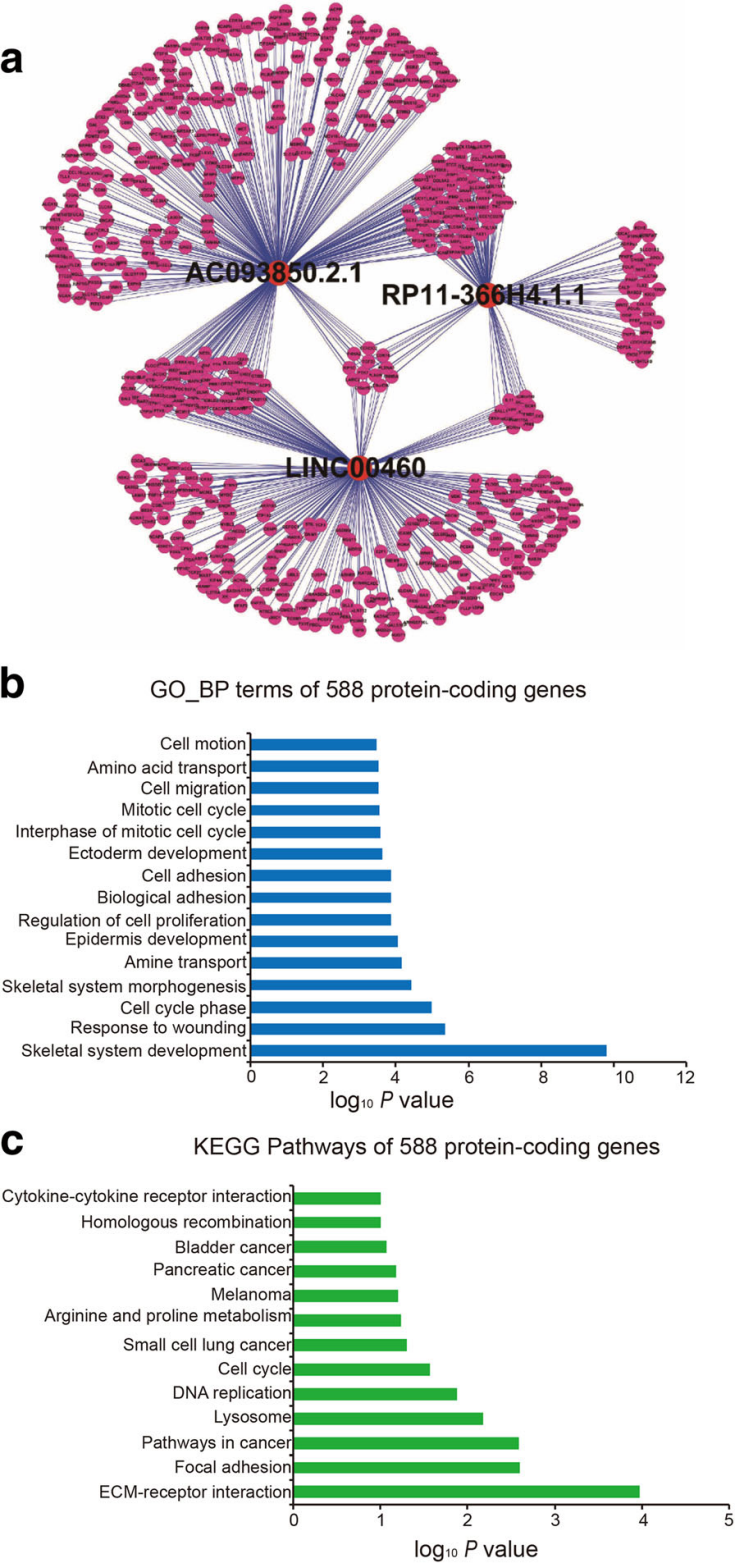
Prediction of the three-lncRNA signature function. **a** 588 protein-coding genes co-expressed with *AC093850.2*, *LINC00460* and *RP11-366H4.1.1* were selected using Pearson correlation coefficients in the lncRNA expression profiles derived from transcriptome RNA-seq analysis of 15 paired ESCC tissues and adjacent normal tissues. Then the association of the three lncRNAs with 588 protein-coding genes was visualized by software Cytoscape_v2.8.3. GO (**b**) and KEGG (**c**) pathway function enrichment analysis for the 588 co-expressed protein-coding genes

To explore the potential biological function of the three-lncRNA signature, we screened protein-coding genes of co-expressed with three lncRNAs, using Pearson correlation coefficients, in the lncRNA expression profiles derived from transcriptome RNA-seq analysis of 15 paired ESCC tissues and adjacent normal tissues [16]. We selected 588 protein-coding genes from the mRNA expression data of the same group of patients, whose expression was highly associated with all or at least one of the three lncRNAs (Pearson correlation coefficient > 0.40, *P* < 0.05). Association of the three lncRNAs with the 588 protein-coding genes was visualized with Cytoscape software v2.8.3. (Fig. 4a). According to the DAVID database (https://david.ncifcrf.gov/) [18], we performed GO (Gene Ontology) and KEGG (Kyoto Encyclopedia of Genes and Genomes) pathway function enrichment analysis for the co-expressed protein-coding genes. GO functional annotation showed that the 588 mRNAs were enriched in 15 GO terms, including skeletal system development, response to wounding and cell cycle phase, and regulation of cell proliferation (Fig. 4b). KEGG analysis suggested that these protein-coding genes took part in ECM-receptor interaction, focal adhesion, pathways in cancer and DNA replication (Fig. 4c). These results indicated that the three lncRNAs might affect tumorigenesis through interacting with protein-coding genes involved in cell development, proliferation adhesion, and other important biological processes.

### *AC093850.2*, *LINC00460* and *RP11-366H4.1.1* lncRNAs promote cancer cell migration and proliferation in vitro

**Fig. 5:**
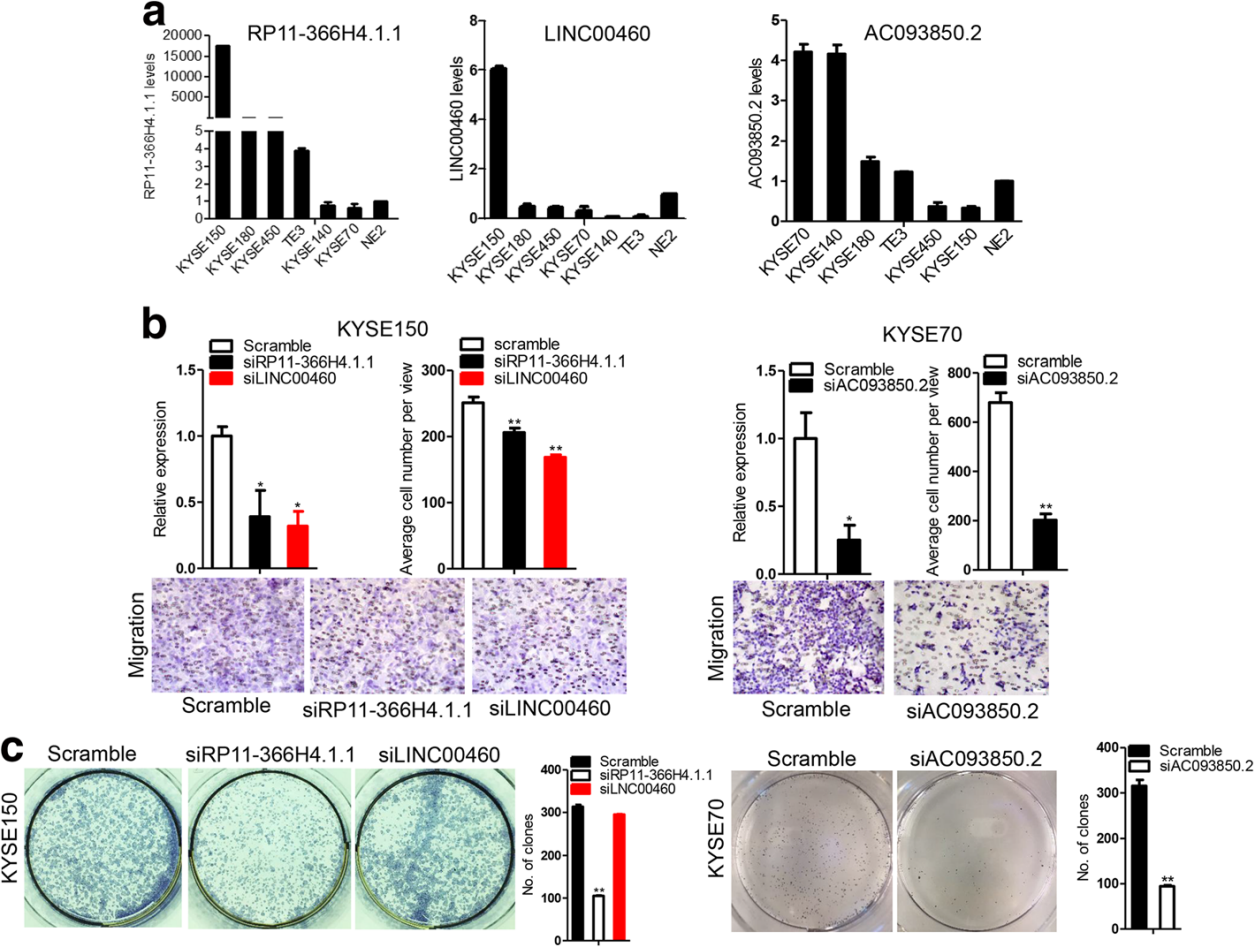
The three lncRNAs promote cancer cell migration and proliferation. **a** Relative expression of the three lncRNAs in various human esophageal cancer cells and immortalized esophageal cells was detected by qRT-PCR. SiRNAs against *RP11-366H4.1.1* or *LINC00460* were transfected into KYSE150 cells and siRNA against *AC093850.2* was transfected into KYSE70 cells. At 24 h post transfection, cells were starved for 12 h in medium without serum and cell migration (**b**) and colony formation assays (**c**) were performed. *P < 0.05, **P < 0.01

We performed qRT-PCR for the three lncRNA expression levels in various human esophageal cancer cells and immortalized esophageal cells and observed that *RP11-366H4.1.1* and *LINC00460* were highly expressed in KYSE150 cells and *AC093850.2* was highly expressed in KYSE70 cells (Fig. 5a). We next individually down-regulated the lncRNAs and found that *RP11-366H4.1.1* knockdown inhibited KYSE150 cell migration and proliferation, whereas knockdown of *LINC00460* only inhibited cell migration, the proliferation of cells was not altered. As for *AC093850.2*, knockdown inhibited both migration and proliferation of KSYE70 cells (Fig. 5b and c). Our results suggest that the three lncRNAs promote cancer cell migration and proliferation.

## Discussion

The seventh edition of the AJCC staging system (p-TNM stage) is the only appropriate reference for predicting the prognosis of patients with ESCC. However, the staging system still needs to be modified in some aspects, and predicting ability needs to be improved further because of the dismal 5-year survival rate [22]. Therefore, there is an immense clinical need for prognostic biomarkers of ESCC. Recent studies demonstrated that the combination of several biomarkers had better predictive ability than individual biomarkers. Different types of prognostic signatures have been identified, including protein-coding gene signatures and non-coding gene signatures. There are protein-coding gene signatures that are highly predictive of ESCC survival in both generation and validation datasets, such as the combination of *EGFR*, *p-Sp1*, and *fascin*; GASC1-targeted genes *PPARG, MDM2, and NANOG*; and a panel of *Annexin II*, *kindlin-2*, and *myosin-9*. However, the protein-coding gene signatures are inadequate to precisely predict clinical outcome of ESCC [11, 23]. MicroRNAs (miRNAs) have their own advantages, for use in testing for specific biomarkers in formalin-fixed, paraffin-embedded (FFPE) tissues and bodily fluids, such as being small in size, containing a stem-loop structure, and being more stable than mRNAs [24]. A recent article reported a four-miRNA signature (composed of hsa-miR-218-5p, hsa-miR-142-3p, hsa-miR-150-5p, and hsa-miR-205-5p) to predict ESCC patient survival [12].

Although miRNA and mRNA prognostic signatures robustly predict the survival of patients with ESCC, lncRNA signatures might help to predict the survival of patients more accurately than previously possible. In the present study, we found another three-lncRNA signature (*AC093850.2*, *LINC00460* and *RP11-366H4.1.1*) in 138 paired ESCC tissues and adjacent normal tissues and robustly predicted the survival of patients. Furthermore, we analyzed the expression of the three lncRNAs in another 18 types of cancers from data derived from the TCGA database and found that only *AC093850.2* and *LINC00460* are associated with breast invasive carcinoma (BRCA) patient survival, whereas *LINC00460* and *RP11-366H4.1.1* are associated with head and neck squamous cell carcinoma (HNSC) patient survival (Additional file 1: Table S3 and Figure S1). This implies the three-lncRNA signature might be specific to ESCC. By the application of the three-lncRNA signature to a test set of 61 patients with ESCC, we observed patients with a low-risk three lncRNA signature in their tumor specimens have longer overall survival than patients with a high-risk signature. The prognostic value of this three-lncRNA signature was further verified in an independent cohort of 119 patients with ESCC.

In the present study, we selected 10 lncRNAs, with high URW-LPE scores obtained from a previous study, for use in further identifying a three-lncRNA signature associated with overall survival and disease-free survival. However, only 3 lncRNAs could actually be confirmed. In the previous study, a lncRNA-PCG co-expression network was constructed using differentially-expressed lncRNAs and known protein coding genes in ESCC [16]. Therefore, based on URWScore, the selected lncRNAs may be associated with cancer cell proliferation, metastasis, differentiation, angiogenesis and survival time of patient with esophageal cancer. In our previous and present study, lncRNAs with a higher URWScore were determined by the comparison of the levels of lncRNAs in paired ESCC samples and adjacent normal tissues, and the survival times of patients were correlated. It is possible that the unconfirmed lncRNAs in this paper are involved in cancer cell differentiation, angiogenesis. Also, as for our algorithms, like other reported algorithms, there is the possibility of potential flaws.

Ten lncRNAs with higher URWScores were selected, and the association of these lncRNAs with the prognosis of OS and DFS of patients with esophageal cancer in the training set were analyzed, resulting in identification of the three lncRNA signature, composed of AC093850.2, LINC00460 and RP11-366H4.1.1, which was verified in test set (*n* = 61) and in an independent cohort (*n* = 119) (Fig. 3). In a previous study, we reported that lncRNA625 (RP11-625H11.2.1) is associated with the prognosis of OS and DFS for patients with stage III esophageal cancer and with lymph node metastasis. In the present study, without considering lymph node metastasis, we analyzed the association of lncRNA625 with prognosis of OS and DFS for patients in the training set (*n* = 77). The results showed that there was no association with prognosis (Fig. 2), suggesting that lncRNA625 is connected with a particular stage of ESCC. As for AC093850.2, LINC00460 and RP11-366H4.1.1, the expression of the three lncRNAs was associated with the prognosis of OS and DFS for patients in the training set (*n* = 77) (Fig. 2). Therefore, it is inappropriate to connect lncRNA625 with AC093850.2, LINC00460 and RP11-366H4.1.1, as a prognostic biomarker signature for OS and DFS of patients with esophageal cancer.

Our studies were distinct from the data published by Li et al. [6]. The main difference between our studies and Li’s was the difference of the algorithms. Based on the lncRNA expression profile microarray, Li et al. adopted a random Forest supervised classification algorithm and a nearest shrunken centroid algorithm and revealed a three-lncRNA signature associated with the survival of esophageal cancer patients. In our previous study, a random walk algorithm was run in a lncRNA-PCG co-expression network constructed using differentially- expressed lncRNAs and known protein coding genes in ESCC. Thus, each lncRNA in the network would be given an URWScore value and lncRNAs with the higher URWScore possess more important biological functions in ESCC [16]. The selected lncRNAs with higher URWScore may be associated with cancer cell proliferation, metastasis, differentiation, angiogenesis and survival time of patient with esophageal cancer. Another difference between our studies with Li’s was that in this study, we reported a novel lncRNA signature for prognostic diagnosis for OS and DFS for patients with esophageal cancer by the comparison of the levels of lncRNAs in paired ESCC samples and adjacent normal tissues, suggesting more potential lncRNAs playing the critical roles in cancer cells.

In conclusion, the three-lncRNA signature is a significant predictor of OS and DFS. This finding might help doctors to individualize prognosis and recurrence. However, the power of the predicted signature needs additional studies involving larger populations for further validation.

## Conclusions

A novel three-lncRNA signature, composed of *RP11-366H4.1.1, LINC00460* and *AC093850.2*, is identified as a potential predictor of overall survival and disease-free survival in patients with esophageal squamous cell carcinoma.

## Additional file

Additional file 1: Table S1. Primers used in this study. **Table S2.** Clinicopathological characteristics of patients with ESCC in the two datasets. **Table S3.** Relationship between lncRNA and survival in different tumor types. **Figure S1.** Kaplan-Meier analysis of OS for expression of *AC093850.2*, *LINC00460* and *RP11-366H4.1.1* in BRCA and HNSC patients. OS, overall survival. (DOCX 397 kb)

## Abbreviations

DFS: Disease-free-survival
ESCC: Esophageal cancer cell
LncRNA: Long non-coding RNA
OS: Overall survival
qRT-PCR: Quantitative RT-PCR
