# Supplementary material for: A three-lncRNA signature predicts overall survival and disease-free survival in patients with esophageal squamous cell carcinoma
Source: BMC Cancer. 2018 Feb 6;18:147. doi: 10.1186/s12885-018-4058-6 (PMC5801805; doi:10.1186/s12885-018-4058-6)
Supplement: Additional file 1: Table S1. — Primers used in this study. Table S2. Clinicopathological characteristics of patients with ESCC in the two datasets. Table S3. Relationship between lncRNA and survival in different tumor types. Figure S1. Kaplan-Meier analysis of OS for expression of AC093850.2, LINC00460 and RP11-366H4.1.1 in BRCA and HNSC patients. OS, overall survival. (DOCX 397 kb) [file 12885_2018_4058_MOESM1_ESM.docx]

**Additional files**

**A three-lncRNA signature predicts overall survival and disease-free survival in patients with esophageal squamous cell carcinoma**

Guo-Wei Huang^1,2,3ψ^, Yu-Jie Xue^1,3ψ^, Zhi-Yong Wu ^1,4ψ^, Xiu-E Xu^1,2^, Jian-Yi Wu^2,3^, Hui-Hui Cao^1,2^, Ying-Zhu^1,2^, Jian-Zhong He^1,2^, Chun-Quan Li^1,2,5^, En-Min Li^2,3^*, Li-Yan Xu^1,2^*

**Additional files**

**Table S1.** Primers used in this study

**Table S2.** Clinicopathological characteristics of patients with ESCC in the two datasets

**Table S3.** Relationship between lncRNA and survival in different tumor types

**Figure S1.** Kaplan-Meier analysis of OS for expression of *AC093850.2*, *LINC00460* and *RP11-366H4.1.1* in BRCA and HNSC patients. OS, overall survival.

| **Table S1.** Primers used in this study | | |
| --- | --- | --- |
| Gene | Forward (5’ to 3’) | Reverse (5’ to 3’) |
| RP11-625H11.2.1 | AGAGACCACCATCAAGGGATAAAAT | GGCTAATAAACAGGGTCTTCAGGT |
| RP11-366H4.1 | CGGTCTAAATGTTGTCTCGCTTCTC | CAGCAATCTGGTCCCTCCTGTCTTC |
| LINC00460 | GCAGAAATCCTCCAGCCCTGTTAGA | GGTGGTTCATCCAGGGTGACTCTTA |
| AC093850.2.1 | AGAGAGGATTCCACTGCGTGTATGT | CTGGGAGGAAGGAACATTGAAGTCT |
| RP13-463N16.6.1 | AGAGAGCAAGAGAGAAGACAGTGGG | AGTGAAGGGCTGAAGGGTTTAGATG |
| RP1-151B14.6.1 | CAGACCAAGGAAAGGCAGCCGAGAC | GTGGCATCTACTTCTTCAGCCTCAA |
| RP11-107M16.2.1 | GTTCCTGATACTGGTTTTTCTACAT | TTTTATTCTCCAAGGTTGTGCTATG |
| LINC01296 | CTCAAACTCCTGGGCTCAAGAAATC | CCATCCCTAAGAATACCCCCACAGT |
| RP11-417E7.1.1 | ATTTGACTCCAGAATGATTTCCAGG | AGGTTTTGTTGCGAAGTGGAATGTG |
| RP11-435D7.3.1 | CTTCCCACATCACTTCCTGACACCT | GAATGAGGTATTTGGGCAGATGTTT |
| β-Actin | AGCGAGCATCCCCCAAAGTT | GGGCACGAAGGCTCATCATT |

| **Table S2. Clinicopathological characteristics of patients with ESCC in the two datasets** | | | | | | |
| --- | --- | --- | --- | --- | --- | --- |
| Clinical and pathological indices | Training set | |  | Test set | | *P-* value |
|  | Case No. | % |  | Case No. | % |  |
| Specimens | 77 |  |  | 61 |  |  |
| Mean age | 58 |  |  | 58 |  |  |
| Age (year) |  |  |  |  |  | 0.681^a^ |
| <58 | 45 | 58.4 |  | 35 | 57.4 |  |
| ≥58 | 32 | 41.6 |  | 26 | 42.6 |  |
| Gender |  |  |  |  |  | 0.689^b^ |
| Male | 58 | 75.3 |  | 48 | 78.7 |  |
| Female | 19 | 24.7 |  | 13 | 21.3 |  |
| Histologic grade |  |  |  |  |  | 0.279^b^ |
| G1 | 17 | 22.1 |  | 8 | 13.1 |  |
| G2 | 55 | 71.4 |  | 46 | 75.4 |  |
| G3 | 5 | 6.5 |  | 7 | 11.5 |  |
| Primary tumor |  |  |  |  |  | 0.968^c^ |
| T1 | 5 | 6.5 |  | 3 | 4.9 |  |
| T2 | 9 | 11.7 |  | 7 | 11.5 |  |
| T3 | 62 | 80.5 |  | 51 | 83.6 |  |
| T4 | 1 | 1.3 |  | 0 | 0 |  |
| Lymph node metastasis (N stage) |  |  |  |  |  | 0.877^c^ |
| N0 | 42 | 54.5 |  | 29 | 47.5 |  |
| N1 | 24 | 31.2 |  | 22 | 36.1 |  |
| N2 | 7 | 9.1 |  | 6 | 9.8 |  |
| N3 | 4 | 5.2 |  | 4 | 6.6 |  |
| pTNM-stage |  |  |  |  |  | 0.423^b^ |
| I | 11 | 14.3 |  | 5 | 8.2 |  |
| II | 36 | 46.8 |  | 27 | 44.3 |  |
| III | 30 | 38.9 |  | 29 | 47.5 |  |

^a^ *t*- test.

^b^ Fisher’s exact test.

^c^ Chi-squared test.

*P* value <0.05 was considered significant.

**Table S3. Relationship between lncRNA and survival in different types of tumor**

| Data Source | Cancer Type | Tumor Samples | | Log-rank *p-*vaule | | |
| --- | --- | --- | --- | --- | --- | --- |
|  |  |  |  | AC093850.2 | LINC00460 | RP11-366H4.1.1 |
| TCGA | Bladder urothelial carcinoma (BLCA) | | 252 | 0.3196 | 0.3024 | 0.9136 |
| TCGA | Brain lower grade glioma (LGG) | | 486 | 0.1578 | 0.3608 | 0.2953 |
| TCGA | **Breast invasive carcinoma (BRCA)** | | 837 | **0.0243** | **0.0102** | 0.0925 |
| TCGA | Cervical squamous cell carcinoma and endocervical adenocarcinoma (CESC) | | 196 | 0.9819 | 0.2650 | 0.2426 |
| TCGA | Colon adenocarcinoma (COAD) | | 157 | na | 0.8832 | na |
| TCGA | Glioblastoma multiforme (GBM) | | 154 | 0.0652 | 0.3922 | 0.2969 |
| TCGA | **Head and neck squamous cell carcinoma (HNSC)** | | 426 | 0.1619 | **0.0002** | **0.0090** |
| TCGA | Kidney renal clear cell carcinoma (KIRC) | | 448 | 0.0910 | **0.0000** | 0.9002 |
| TCGA | Kidney renal papillary cell carcinoma (KIRP) | | 198 | 0.1432 | 0.5471 | **0.0098** |
| TCGA | Liver hepatocellular carcinoma (LIHC) | | 200 | 0.9426 | 0.8978 | 0.4610 |
| TCGA | Lung adenocarcinoma (LUAD) | | 488 | 0.3226 | 0.3385 | 0.1639 |
| TCGA | Lung squamous cell carcinoma (LUSC) | | 220 | 0.0639 | 0.7213 | 0.1158 |
| TCGA | Ovarian serous cystadenocarcinoma (OV) | | 412 | 0.6058 | 0.9886 | 0.1708 |
| TCGA | Prostate adenocarcinoma (PRAD) | | 374 | 0.3865 | 0.5271 | na |
| TCGA | Skin cutaneous melanoma (SKCM) | | 226 | na | na | na |
| TCGA | Stomach adenocarcinoma (STAD) | | 285 | 0.2806 | 0.6090 | 0.3995 |
| TCGA | Thyroid carcinoma (THCA) | | 497 | 0.3865 | 0.1933 | 0.8638 |
| TCGA | Uterine corpus endometrioid carcinoma (UCEC) | | 316 | na | 0.1758 | na |

**
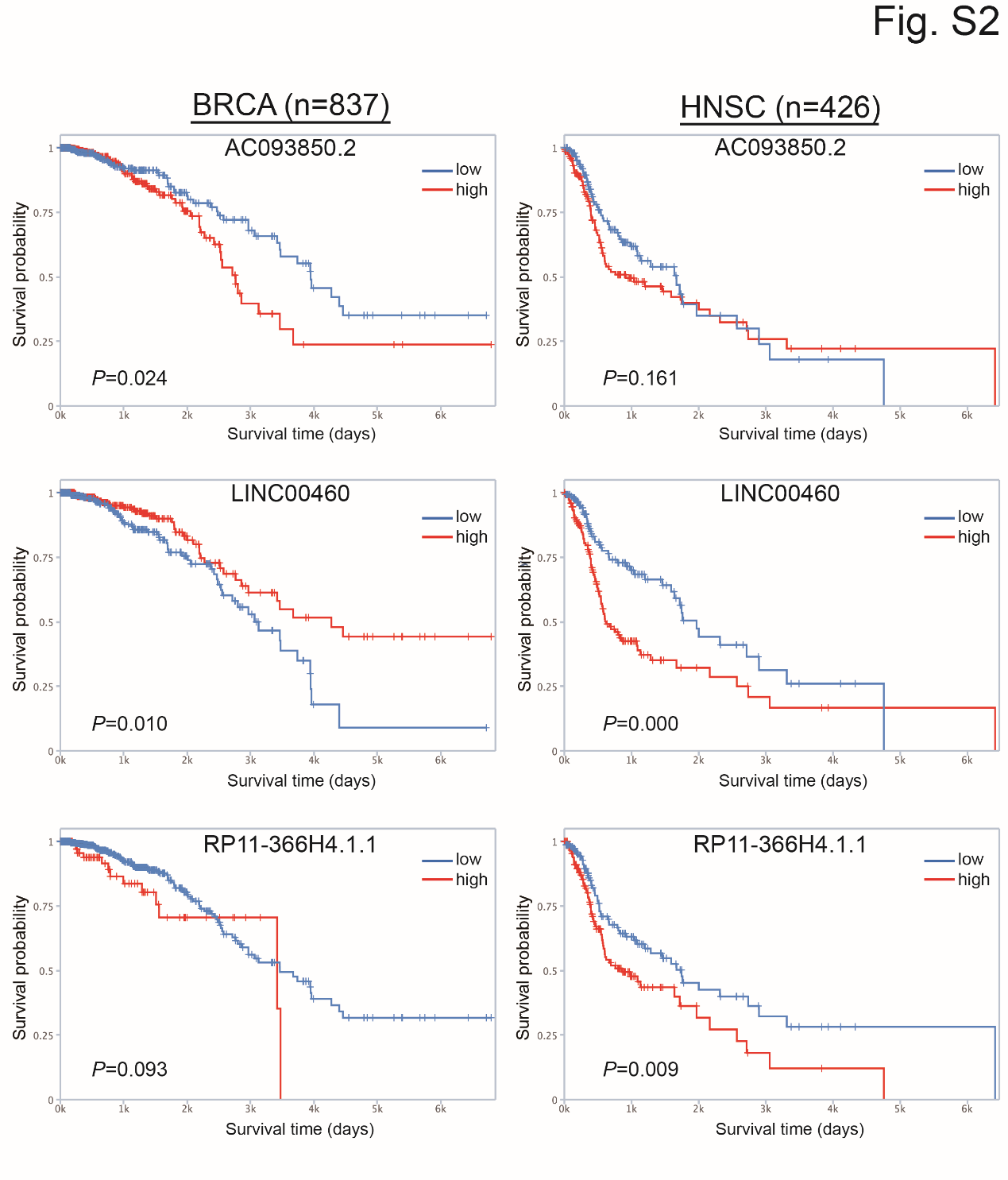
**

**Figure S1.** Kaplan-Meier analysis of OS for expression of AC093850.2, LINC00460 and RP11-366H4.1.1 in BRCA and HNSC patients. OS, overall survival.
